# Altering the selection capabilities of common cloning vectors via restriction enzyme mediated gene disruption

**DOI:** 10.1186/1756-0500-6-85

**Published:** 2013-03-06

**Authors:** Sam Manna, Ashley Harman, Jessica Accari, Christian Barth

**Affiliations:** 1 Department of Microbiology, La Trobe University, Melbourne, VIC 3086, Australia

**Keywords:** Molecular cloning, Gene disruption, Selection of transformants, Antibiotic resistance

## Abstract

**Background:**

The cloning of gene sequences forms the basis for many molecular biological studies. One important step in the cloning process is the isolation of bacterial transformants carrying vector DNA. This involves a vector-encoded selectable marker gene, which in most cases, confers resistance to an antibiotic. However, there are a number of circumstances in which a different selectable marker is required or may be preferable. Such situations can include restrictions to host strain choice, two phase cloning experiments and mutagenesis experiments, issues that result in additional unnecessary cloning steps, in which the DNA needs to be subcloned into a vector with a suitable selectable marker.

**Results:**

We have used restriction enzyme mediated gene disruption to modify the selectable marker gene of a given vector by cloning a different selectable marker gene into the original marker present in that vector. Cloning a new selectable marker into a pre-existing marker was found to change the selection phenotype conferred by that vector, which we were able to demonstrate using multiple commonly used vectors and multiple resistance markers. This methodology was also successfully applied not only to cloning vectors, but also to expression vectors while keeping the expression characteristics of the vector unaltered.

**Conclusions:**

Changing the selectable marker of a given vector has a number of advantages and applications. This rapid and efficient method could be used for co-expression of recombinant proteins, optimisation of two phase cloning procedures, as well as multiple genetic manipulations within the same host strain without the need to remove a pre-existing selectable marker in a previously genetically modified strain.

## Background

Molecular cloning is a process involving the incorporation of a copy of a gene or gene fragment into a plasmid vector [1]. This process allows the characterisation of genes and their associated gene products from any organism. Hence, the ability to clone genes for their characterisation completely revolutionised molecular biology and our understanding of cell biology.

Most cloning vectors are taken up, stably maintained and efficiently replicated in *Escherichia coli*. As a result, *E. coli* is commonly used as a tool for cloning. Given the low efficiency of vector transformation, one important step in the cloning process is the selection of cellular clones carrying vector DNA, termed transformants [2]. Selection will only allow cells carrying vector DNA to grow, while untransformed cells are inhibited or killed due to the presence of a selective agent [3]. For molecular cloning in *E. coli,* selection of transformants carrying vector DNA often involves using antibiotics which are supplemented into the growth medium. This is because the vector contains a gene encoding resistance to a particular antibiotic, termed a selectable marker gene [2,3]. Due to this resistance being vector-encoded, the resistance phenotype is only conferred to transformants carrying the vector. Additionally, the selective pressure placed upon transformants by using selective agents, forces the *E. coli* host to maintain and replicate the vector. Given that maintenance of a vector, which provides no advantage in a given environment, can place transformants at a growth disadvantage, a lack of selection can promote vector loss [4].

For the selection of vectors in *E. coli*, there is an array of selective agents and associated selectable marker genes encoded in these vectors. While such agents include ionic heavy metals such as mercury, it is antibiotics that are most commonly used for selecting *E. coli* transformants [4]. One of the most common antibiotics utilised is ampicillin. Vector-encoded ampicillin resistance genes usually encode β-lactamases, which enzymatically inactivate ampicillin [3,5,6]. While ampicillin selection provides a number of advantages, there are often circumstances in which selection with a different antibiotic is desired. For example, β-lactamases are produced at substantial levels, and are secreted into the medium. Prolonged incubation of these cells results in the inactivation of all ampicillin in the medium [3,4]. Thus, after a significant period of time into incubation, cells will continue growing in the absence of selective pressure. In transformation experiments, this will lead to the formation of ampicillin sensitive satellite colonies.

Additionally, maintaining two different vectors within one host cell often requires differing selectable markers for each vector. If both vectors confer resistance to the same antibiotic, the selective pressure will often not be sufficient for the host to maintain both replicons. Moreover, many host strains have been genetically modified and as a result, carry resistance to specific antibiotics. This innate resistance means that vectors conferring resistance to the same antibiotic are not suitable for this strain. This presents an issue when such restrictions mean the researcher is forced to choose either another strain or another vector, which may be less suitable for the experiment of interest. Additionally, other genetic manipulation methods, such as mutagenesis or conjugation experiments, also require the use of selectable markers for which limited choices are also available. Furthermore, difficulties can also be encountered when a cloned DNA sequence of interest is in a vector that does not carry a suitable selectable marker and thus, needs to be subcloned into a different vector. Thus, the ability to rapidly change the selectable marker capabilities of an existing vector or construct of interest is a highly desirable feature in many aspects of genetic research, such as improving molecular cloning and transformant selection methods.

Here, we report a cloning procedure that involves altering the selectable marker gene of a vector of interest by introducing another. This is performed using restriction enzyme mediated gene inactivation in which the initial vector encoded selectable marker gene is inactivated by cloning another selectable marker gene into the original open reading frame (ORF). Using this process to expand selectable marker choice has a number of potential applications and provides multiple benefits for many procedures including cloning, mutagenesis, host strain choice and vector choice.

## Methods

### PCR amplification of tetracycline and kanamycin selectable marker genes

The *tetA* gene cassette was amplified using pLAFR1 vector as a template with forward primer 5′-CCATGGCTGCAGAGTACTGTTTCCACGATCAGCGATCGGCT CG-3′ and reverse primer 5′-CCATGGCTGCAGAGTACTGGCACGGATCACTGTATTCGGCTGC-3′. These primers had restriction sites for *Nco*I, *Pst*I and *Sca*I incorporated for cloning purposes. The *kan*^*R*^ gene cassette was amplified using the pZErO-2 vector as template with forward primer 5′-AGTACTCAAACTGGAACAACACTCAACCCTATCG-3′ and reverse primer 5′-AGTACTCACCTAGATCCTTTTCACGTAGAAAGCC-3′. These primers had restriction sites for *Sca*I incorporated for cloning purposes. PCR reactions were performed under the following cycling conditions; 94°C for 5 min and then 40 cycles of 94°C for 1 min, 60°C for 30 seconds, 72°C for 1 min followed by a final extension at 72°C for 11 min.

### Cloning of tetracycline and kanamycin selectable marker genes

The *tetA* and *kan*^*R*^ gene cassettes have been amplified by PCR using gene specific primers as described above. The amplification products were cloned into the phase I vector pCR®2.1-TOPO® (Invitrogen) according to the manufacturer’s instructions. The phase I constructs and the phase II vectors were then digested with the relevant restriction enzyme in order to clone into the ORF of the phase II vector’s selectable marker gene. The gel purified insert and vector were then used in a ligation reaction using T4 DNA ligase (Promega). Cohesive end ligations were performed at 16°C/23°C cycling conditions for 12 hours, while blunt end ligations were performed at 15°C for 12 hours. Aliquots of the ligation reaction were then electroporated into *E. coli* DH5α or TOP10 cells at a capacitance of 25 μF at 2.5 kV with a resistance of 200 Ω. The transformation was plated onto Luria Bertani (LB) agar supplemented with the antibiotic to which the insert confers resistance (tetracycline or kanamycin, 5 μg/ml and 25 μg/ml, respectively).

### Viable transformant counts

In order to determine the number of viable cells and their ability to grow in the presence of different antibiotics, overnight cultures were serially diluted to 10^-7^. The *E. coli* transformation mixtures of interest were then plated out onto LB agar containing either no antibiotic, ampicillin (100 μg/ml), kanamycin (25 μg/ml) or tetracycline (5 μg/ml). Viable transformant counts were calculated based on the number of colonies obtained on duplicate plates of the same dilution within a statistically valid range.

### Southern blot hybridisation

Vector DNA was linearised and run on a 1% (w/v) agarose gel. The gel was washed in depurination (0.25 M HCl), neutralisation (0.5 M Tris–HCl, pH 7.0) and denaturation (1.5 M NaCl, 0.4 M NaOH) solutions, and the DNA was transferred to a nylon membrane (Hybond). After the membrane was baked at 80°C for 2 hours, the membrane was prehybridised in hybridisation buffer (25% 20 × SSC [3 M NaCl, 0.3 M Tri-sodium citrate], 50% formamide, 0.1% N-lauroyl-sarcosine, 0.02% SDS, 2% blocking solution) at 42°C overnight. The pBR328 vector DIG labelled control probe provided with the DIG labelling and detection kit (Roche) was used as a probe to detect vector DNA and was added to the hybridisation buffer at 25 ng/ml. After incubating overnight, DIG detection was performed using colourimetric detection according to the manufacturer’s instructions.

## Results

### Restriction enzyme mediated gene disruption for changing selectable markers

**Figure 1:**
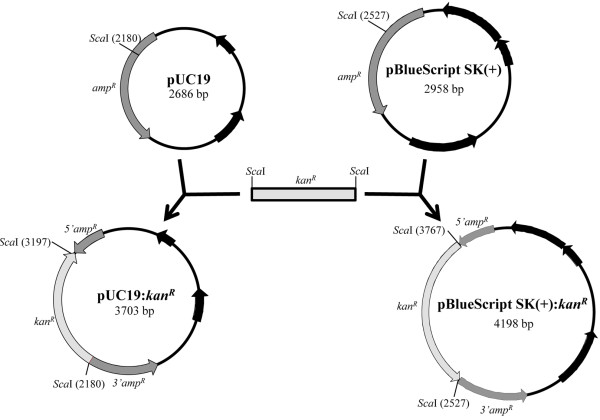
**Strategies employed for cloning a kanamycin resistance cassette (*kan***^***R***^**) via selectable marker restriction enzyme mediated gene disruption.** The cassette was cloned into the unique *Sca*I site present in the *amp*^*R*^ gene of pUC19 and pBlueScript SK+. Relevant restriction sites and features of each vector are indicated.

**Figure 2:**
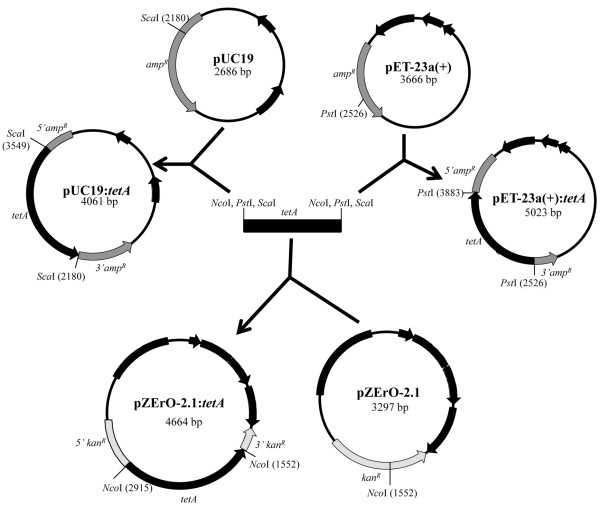
**Strategies employed for cloning a tetracycline resistance cassette (*tetA*) via selectable marker restriction enzyme mediated gene disruption.** The cassette was cloned into the resistance genes of pUC19, pZErO-2 and pET23a using unique restriction sites located in the selectable marker of each vector. Relevant restriction sites and features of each vector are indicated.

In order to establish a simple method for modifying the selectable marker gene in commonly used *E. coli* vectors, four different vectors and two resistance cassettes conferring resistance to kanamycin and tetracycline were used (Table 1). Two of the vectors, pUC19 [7-9] and pZErO-2 (Invitrogen), are commonly used for phase I cloning. The vector pBlueScript SK(+) (Stratagene), is a phagemid, which is widely used for *in vitro* transcription of cloned DNA [10]. The final source vector used, pET23a (Novagen) is an *E. coli* expression vector which was employed to further demonstrate the versatility and potential applications of this selection modification method. The cloning strategies are outlined in Table 1 and in Figures 1 and 2.

**Table 1:** Vectors used for restriction enzyme mediated selectable marker gene disruption

**Original Vector (and source)**	**Phenotype of original selectable marker**	**Cloning restriction site**	**Phenotype of cloned selectable marker**	**Construct name**
pUC19 (New England Biolabs)	amp^R^	*Sca*I	kan^R^	pUC19:*kan*^*R*^
pUC19 (New England Biolabs)	amp^R^	*Sca*I	tet^R^	pUC19:*tetA*
pZErO-2 (Invitrogen)	kan^R^	*Nco*I	tet^R^	pZErO-2:*tetA*
pBlueScript SK + (Stratagene)	amp^R^	*Sca*I	kan^R^	pBlueScript SK+: *kan*^*R*^
pET23a (Novagen)	amp^R^	*Pst*I	tet^R^	pET23a:*tetA*

amp^R^ : Ampicillin resistance, kan^R^ : Kanamycin resistance, tet^R^ : Tetracycline resistance.

Once a suitable restriction site within the existing antibiotic resistance gene was identified, gene specific primers were designed to PCR amplify the *tetA* and *kan*^*R*^ gene cassettes (conferring resistance to tetracycline and kanamycin, respectively) and their associated regulatory elements, using other vectors containing these genes as templates. The tetracycline resistance locus in pLAFR1 consists of two genes, *tetA* and *tetR*[11-13]. The *tetA* gene encodes an efflux pump responsible for the resistance phenotype, while *tetR* encodes a regulatory repressor protein [13-15]. As negative regulation was not required, only the *tetA* gene was amplified. The *kan*^*R*^ gene, amplified from pZErO-2, encodes a aminophosphotransferase, APH(3′)-II isolated from transposon tn*5*, which confers resistance to kanamycin via enzymatic inactivation [2,16].

### Creation and isolation of transformants carrying vectors with the new selectable marker gene

Following amplification and cloning of the *tetA* and *kan*^*R*^ cassettes into pCR®2.1-TOPO® (Invitrogen) phase I vector (data not shown), the cloned resistance cassettes and target vectors of interest were digested with the suitable restriction enzyme (Table 1). After ligation and transformation of *E. coli*, cultures were plated onto LB agar supplemented with the antibiotic for which the insert encodes resistance. This allowed for selection of the transformants carrying the construct of interest. Transformants carrying empty, recircularised vectors were therefore selected against on this medium. Both the *tetA* and *kan*^*R*^ resistance cassettes were successfully cloned into the *amp*^*R*^ gene of pUC19 (Figures 1 and 2). The *tetA* resistance cassette was also cloned into the *kan*^*R*^ and *amp*^*R*^ genes of pZErO-2 and pET23a, respectively (Figure 2). Lastly, the *kan*^*R*^ resistance cassette was also cloned into the *amp*^*R*^ gene of pBlueScript (Figure 1).

### Cloning a new resistance cassette into the original selectable marker gene of any vector abolishes the original resistance phenotype and subsequently confers resistance to a different antibiotic

**Figure 3:**
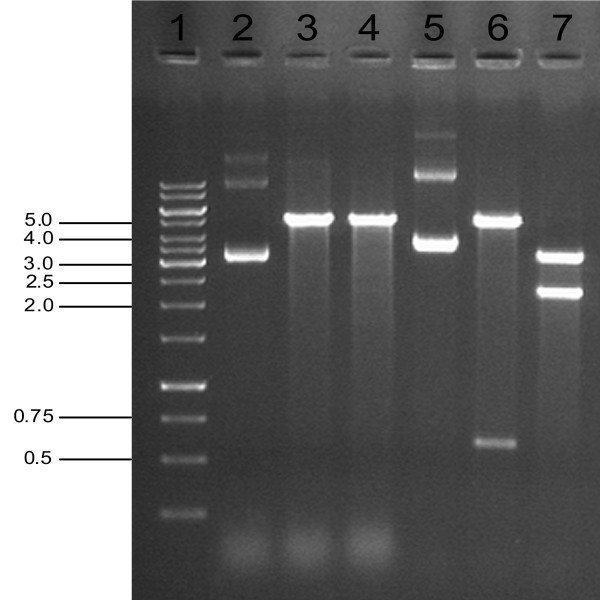
**Restriction digest analysis of the vector pET23a:*tetA *carrying a foreign DNA segment.** Cloning of a 590 bp foreign DNA was performed using *Sac*I and *Hin*dIII restriction sites located within the multiple cloning site of pET23a:*tetA*. Lane 1: 1 kb gene ruler DNA ladder (sizes as indicated in kb); lane 2: pET23a:*tetA* vector undigested; lane 3: pET23a:*tetA*, digested with *Sac*I and *Hin*dIII (5.01 kb); lane 4: pET23a:*tetA*, digested with *Eco*RV, a restriction site located within the *tetA* gene (5.02 kb); lane 5: pET23a:*tetA* vector carrying a 590 bp cloned DNA segment, undigested; lane 6: pET23a:*tetA* vector carrying a 590 bp cloned DNA segment, digested with *Sac*I and *Hin*dIII (0.59, 5.01 kb); lane 7: pET23a:*tetA* vector containing cloned DNA, digested with *Eco*RV, a restriction site located within both the *tetA* gene and the cloned DNA (3.30, 2.30 kb).

In order to determine if restriction enzyme mediated gene disruption inactivated the original vector resistance ORF, viable transformant counts were performed for *E. coli* transformants carrying either the original vector or the construct containing the new selectable marker. For all five constructs, the transformants only grew in the presence of the antibiotic for which the cloned gene encodes resistance to (Tables 2, 3, 4). This indicates that insertion of a new selectable marker gene into the original resistance ORF of a vector confers resistance to a new antibiotic, and that the vector no longer provides resistance to the original antibiotic as a result of gene disruption. This phenotype was observed for all transformants carrying any of the five constructs, while transformants carrying the empty, original vector displayed the opposite resistance profile. It is also important to note that the practical value of the constructs as cloning vectors remained unaffected, since this method only involved disruption of the selectable marker gene, leaving the multiple cloning sites and other regulatory features of these vectors intact. This was demonstrated by the successful cloning of a foreign DNA sequence (590 bp in size) into the *Sac*I and *Hin*dIII restriction sites in the multiple cloning site of the vector pET23a:*tetA* (Figure 3).

**Table 2:** **Viable transformant counts of *E. coli *transformants carrying vectors with an undisrupted or disrupted ampicillin selectable marker**

	**Viable transformant count (cfu/ml) on medium**		
**Transformant**	**LB**	**LB + Amp**	**LB + Tet**
pUC19	2.22 × 10^9^	2.25 × 10^9^	0
pUC19:*tetA*	4.2 × 10^9^	0	2.85 × 10^9^
pET23a	2.85 × 10^9^	2.39 × 10^9^	0
pET23a:*tetA*	2.03 × 10^9^	0	2.11 × 10^9^

Disruptants were created by cloning a tetracycline selectable marker gene into a unique restriction site in the vector encoded ampicillin resistance gene. Transformants were plated onto LB medium supplemented with either ampicillin or tetracycline. cfu; colony forming unit.

**Table 3:** **Viable transformant counts of *E. coli *transformants carrying vectors with an undisrupted or disrupted kanamycin selectable marker**

	**Viable transformant count (cfu/ml) on medium**		
**Transformant**	**LB**	**LB + Kan**	**LB + Tet**
pZErO	2.05 × 10^9^	1.35 × 10^9^	0
pZErO:*tetA*	1.06 × 10^7^	0	1.07 × 10^7^

Disruptants were created by cloning a tetracycline selectable marker gene into a unique restriction site in the vector encoded kanamycin resistance gene. Transformants were plated onto LB medium supplemented with either kanamycin or tetracycline. cfu; colony forming unit.

**Table 4:** **Viable transformant counts of *E. coli *transformants carrying vectors with an undisrupted or disrupted ampicillin selectable marker**

	**Viable transformant count (cfu/ml) on medium**		
**Transformant**	**LB**	**LB + Amp**	**LB + Kan**
pUC19	2.22 × 10^9^	2.25 × 10^9^	0
pUC19:*kan*^*R*^	6.7 × 10^8^	0	7.3 × 10^8^
pBlueScript	2.87 × 10^9^	2.81 × 10^9^	0
pBlueScript:*kan*^*R*^	2.05 × 10^9^	0	1.78 × 10^9^

Disruptants were created by cloning a kanamycin selectable marker gene into a unique restriction site in the vector encoded ampicillin resistance gene. Transformants were plated onto LB medium supplemented with either ampicillin or kanamycin. cfu; colony forming unit.

### Determining relative vector copy number in co-transformed cells for expression vectors

**Figure 4:**
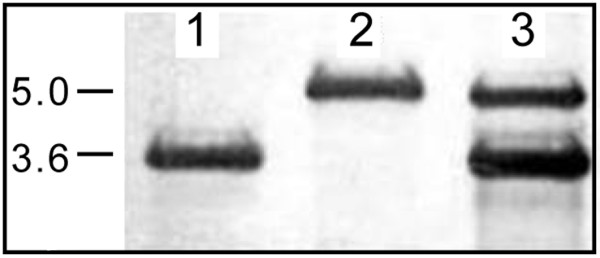
**Analysis of relative vector copy number of pET23a and pET23a:*tetA *in *E. coli *co-transformed cells.** The blot displays signals for *Eco*RI linearised vectors from individual transformants carrying the original pET23a vector (lane 1), the pET23a:*tetA* (lane 2), or vector DNA from co-transformants carrying both pET23a and pET23a:*tetA* vectors (lane 3).

In order to investigate the suitability of pET23a:*tetA* for expression experiments in *E. coli*, the stability and relative copy number of pET23a and pET23a:*tetA* were determined in transformants individually, as well as in cells co-transformed with both vectors. Southern blot hybridisation analysis demonstrated that vector copy number from cells carrying either pET23a or pET23a:*tetA* were similar (Figure 4). When both vectors were introduced into the same host, they were both maintained under selective pressure despite the incompatibility of their replication origins, and the cells efficiently grew in the presence of both ampicillin and tetracycline (data not shown). However, pET23a:*tetA* displayed a reduced copy number compared to pET23a, which only seemed to occur in the presence of pET23a.

## Discussion

### Restriction enzyme mediated gene disruption inactivates the function of the original selectable marker and provides a novel selection system

Selection of bacterial transformants is an important step in both the cloning of DNA and for the maintenance of vector DNA in bacterial cells. The type of selectable marker present in a particular vector is often an important consideration in multiple aspects of research. On many occasions, there is a lack of choice when choosing a vector and its selection capabilities. Here, we have demonstrated a procedure to easily change the selectable marker gene in desired cloning vectors by restriction enzyme mediated gene disruption.

Restriction enzyme mediated gene disruption is a well established method in molecular cloning and is primarily used to distinguish between *E. coli* transformants carrying an empty vector and those carrying a vector with a cloned insert. The best known example of this is blue-white screening, which involves cloning the DNA of interest into the *Lac*Zα gene encoding the α subunit of the β-galactosidase enzyme [17]. Transformants carrying the empty vector (and an intact *Lac*Zα gene) produce blue colonies in the presence of X-gal, the β-galactosidase substrate. On the other hand, in transformants carrying vectors with cloned inserts, the *Lac*Zα gene has been disrupted and the colonies remain white [18,19].

Restriction enzyme mediated gene disruption was also traditionally used in the inactivation of selectable marker genes for identifying transformants carrying vectors with the cloned insert. The pACYC vectors contain multiple antibiotic resistance genes within the one vector and one of the resistance genes contains a cloning site. Hence, cloning DNA into these vectors inactivated one of the antibiotic resistance genes and therefore transformants carrying the cloned DNA would be sensitive to this antibiotic, while remaining resistant to the second antibiotic conferred by the intact resistance gene [17,20].

Here, we have utilised the same principle, but instead of using it to screen for transformants carrying vectors with cloned inserts, we have used it to change the selection capabilities of vectors. Insertion of either the *tetA* or *kan*^*R*^ genes into the original selectable marker of multiple vectors was found to abolish the original resistance phenotype conferred by the original ORF and provides resistance to another antibiotic that was encoded by the cloned DNA (Tables 2, 3, 4).

### Significance of selectable marker restriction enzyme mediated gene disruption

Restriction enzyme mediated gene disruption to change the antibiotic resistance gene in a vector provides multiple advantages, especially due to the versatility and simplicity of the process. We have shown that this method can be used to easily change the selection profile of commonly used *E. coli* vectors, including both cloning and expression vectors, thereby increasing the availability of suitable vectors. The method is simple, as any restriction site of interest can be chosen as long as it is unique and located within the original antibiotic resistance ORF, and we have provided a list of commonly used vectors and suitable restriction sites for selectable marker gene disruption in Table 5.

**Table 5:** Commonly used vectors and unique restriction sites suitable for selectable marker restriction enzyme mediated gene disruption

**Vector**	**Selectable marker**	**Source**	**Unique restriction sites in selectable marker**
pUC18, pUC19	amp^R^	New England Biolabs	*Xmn*I, *Sca*I
pBlueScript	amp^R^	Stratagene	*Sca*I
pZErO	kan^R^	Invitrogen	*Nco*I
pBR322	amp^R^ tet^R^	New England Biolabs	*Sca*I, *Pvu*I, *Pst*I *Eco*RV, *Bam*HI, *Sph*I, *Sal*I
pACYC177	amp^R^ kan^R^	New England Biolabs	*Sca*I, *Pst*I, *Bgl*I *Cla*I, *Hind*III
pACYC184	cam^R^ tet^R^	New England Biolabs	*Eco*RI, *Nco*I *Eco*RV, *Bam*HI, *Sph*I, *Sal*I

*amp*^*R*^ : ampicillin resistance, *kan*^*R*^ : kanamycin resistance, *tet*^*R*^ : tetracycline resistance, *cam*^*R*^ : chloramphenicol resistance.

The method is also simple, as it requires only one additional cloning step to create the desired vector, and the screening process for transformants of interest is rapid and efficient. This is because construct carrying transformants can be isolated on media supplemented with the antibiotic for which resistance is encoded for by the cloned gene. For example, pUC19 transformants can grow on ampicillin but cannot grow on tetracycline. On the other hand, pUC19 transformants carrying the *tetA* cassette display the opposite growth characteristics (Table 2). Therefore, all transformants obtained on tetracycline plates were highly likely to contain vectors with the *tetA* cassette integrated into the *amp*^*R*^ gene. Thus this method can be applied to vectors to be used in downstream cloning applications. Additionally, it can also be used to modify the selectable marker of an existing construct containing cloned DNA, rather than having to repeat the cloning of the DNA of interest into a different vector with suitable selection capabilities, which can be tedious and time consuming.

Although ampicillin is widely used in cloning as a selective agent, there are multiple disadvantages of working with ampicillin. Ampicillin is inactivated by β-lactamases, enzymes encoded by ampicillin resistance genes [3,5,6]. Given that they are expressed at high levels and are secreted by cells, the medium is quickly exhausted of ampicillin during incubation [3,4]. This results in continual growth of bacterial cultures in the absence of selection, which can result in loss of vector DNA and ampicillin sensitive satellite colonies arising in transformations. Additionally, high concentrations of ampicillin (~100 μg/ml) are often required and ampicillin solutions cannot be stored for extended periods of time. In contrast, tetracycline resistance conferred by *tetA* is based on an efflux pump mechanism [13-15] and as a result, tetracycline is never inactivated but present during the entire incubation, maintaining selective pressure. Also, in comparison to ampicillin, tetracycline usually requires much lower concentrations (~5 μg/ml). As a result, tetracycline may be a more attractive antibiotic for use in molecular cloning over ampicillin. Therefore, the pUC19:*tetA* construct created via restriction enzyme mediated gene disruption provides a beneficial vector for use in molecular cloning over its parental vector (pUC19).

### Applications of selectable marker restriction enzyme mediated gene disruption in molecular cloning and studying cell biology

The versatility of selectable marker gene disruption via cloning also means that there are a number of applications to this method for studying cell biology. Firstly, changing the selection capabilities of a vector can significantly improve and facilitate the cloning of a DNA fragment of interest. If the selectable marker gene of either the phase I or phase II vector is changed via restriction enzyme mediated gene disruption, the two vectors now confer different resistance phenotypes. As a result, subcloning from the phase I to the phase II vector is possible without the need to purify the insert from the phase I backbone. Although the ligation reaction will now also include the phase I vector as well as the insert and phase II vector, selection of the transformants can be based on the resistance conferred by the phase II vector. Thus, transformants containing any recombinant form of the phase I vector will be selected against and will not grow on the medium. This improves cloning efficiency and allows evasion of purification procedures, such as gel extraction, which can degrade the ends of the insert as well as the unavoidable contamination of agarose which, can contain DNA ligase inhibiting components [21,22]. Other purification methods such as phenol chloroform extraction can leave traces of phenol, which can also interfere with subsequent ligation reactions. Additionally, the purification methods do not always completely remove the phase I vector backbone, and phase I vector contamination in phase II ligations and transformations will make screening for recombinants more laborious, if the phase I vector is not selected against. For example, if a DNA fragment of interest was subcloned from pUC19 (*amp*^*R*^) to pET23a (*amp*^*R*^), pUC19 contamination will lower the chance of obtaining the desired construct. However, this can be avoided if pET23a:*tetA* (Figure 2) was used instead, as selection would be performed based on tetracycline resistance instead of ampicillin resistance.

While vector-based antibiotic resistance is most commonly used to isolate and maintain vector constructs in *E. coli*, it also forms the basis for the generation of bacterial strains via insertional mutagenesis. However, many *E. coli* strains used for mutagenesis studies already have resistance to particular antibiotics [18], and other *E. coli* strains, as well as many other bacteria, have innate resistance to specific antibiotics [3,18]. In these circumstances, the choice of selectable markers and suitable vectors is greatly restricted. To overcome this, restriction enzyme mediated gene disruption can be used to change the selection phenotype conferred by the particular vector being used, by choosing a selectable marker to which the strain of interest is not already resistant. Similarly, this method also allows for successive gene manipulations in the same strain and facilitates the isolation of double mutants making the need for methods such as the *cre-lox* system for reusing the same selectable marker unnecessary. While the *cre-lox* system can provide benefits for the removal and reuse of selectable markers in particular strains [23], using restriction enzyme mediated gene disruption offers a simpler and less time consuming solution for multiple gene manipulations within the same strain.

Most vectors used for the heterologous expression of recombinant proteins in *E. coli* possess ampicillin resistance genes [24]. In fact, there are very few *E. coli* expression vectors that have a resistance gene other than ampicillin. This can present an issue given the disadvantages of working with ampicillin discussed above, but can also limit the choice of vectors for co-expression studies. Using selectable marker restriction enzyme mediated gene disruption, we modified the selectable marker of pET23a (Novagen), a commonly used expression vector. This involved inactivation of the *amp*^*R*^ gene, via the successful introduction of the *tetA* gene cassette, which was confirmed at the phenotypic level (Table 2). Similar to the original vector, the pET23a:*tetA* construct can now be used for cloning and subsequent protein expression, and we also demonstrated that both vectors are maintained and transmitted to daughter cells when co-transformed in the same host, despite the incompatibility of their replication origins. Using this method to change the selectable marker gene of an expression vector therefore allows the co-expression of two recombinant proteins within the one host cell as both vectors can be maintained under selective pressure, regardless if the replication origins of the two vectors are incompatible. Co-expression of recombinant proteins in the same host is important for the study of protein-protein interactions or the co-expression of molecular chaperones to improve the folding and solubility of a particular recombinant protein [25,26]. Using restriction enzyme mediated gene disruption to change the selectable marker of protein expression vectors allows such applications to be improved.

## Conclusions

We have described a method in which the basic principles of cloning have been utilised to change the antibiotic resistance phenotype conferred by an *E. coli* vector by cloning a different resistance gene into the original ORF. This simple and rapid method has a number of advantages including high versatility and options for researchers. It also has applications in cloning, mutagenesis studies and heterologous protein expression. Therefore, as a method, selectable marker restriction enzyme mediated gene disruption can provide a significant contribution to studying molecular biology in bacteria as well as in other organisms.

## Competing interests

The authors declare that they have no competing interests.

## Authors’ contributions

SM created all pCR®2.1-TOPO®, pUC19:*tetA* and pET23a:*tetA* constructs, as well as the southern blot and viable transformant count procedures, in addition to writing the manuscript. AH created the pUC19:*kanR* and pBlueScript:*kanR* constructs and drafted the manuscript. JA created the pZErO2:*tetA* construct and drafted the manuscript. CB contributed to the adaptation of the method for selectable markers, laboratory troubleshooting of the procedure, in addition to the writing and drafting of the manuscript. All authors have read and approved the manuscript.

## Authors’ information

SM, AH and JA were the recipients of Australian Postgraduate Awards.
